# Gene model for the ortholog of *dock* in *Drosophila ananassae*

**DOI:** 10.17912/micropub.biology.001025

**Published:** 2025-09-14

**Authors:** Anne E. Backlund, Tamica D’Souza, Jaskamaldip Kaur, Anya Goodman, James J. Youngblom, Chinmay P. Rele, Laura K Reed

**Affiliations:** 1 The University of Alabama, Tuscaloosa, AL USA; 2 California Polytechnic State University, San Luis Obispo, CA USA; 3 California State University Stanislaus, Turlock, CA USA

## Abstract

Gene model for the ortholog of *dreadlocks* ( *dock* ) in the May 2011 (Agencourt dana_caf1/DanaCAF1) Genome Assembly (GenBank Accession: GCA_000005115.1 ) of *Drosophila ananassae* . This ortholog was characterized as part of a developing dataset to study the evolution of the Insulin/insulin-like growth factor signaling pathway (IIS) across the genus *Drosophila* using the Genomics Education Partnership gene annotation protocol for Course-based Undergraduate Research Experiences.

**Figure 1. Genomic neighborhood and gene model for dock in Drosophila ananassae:**
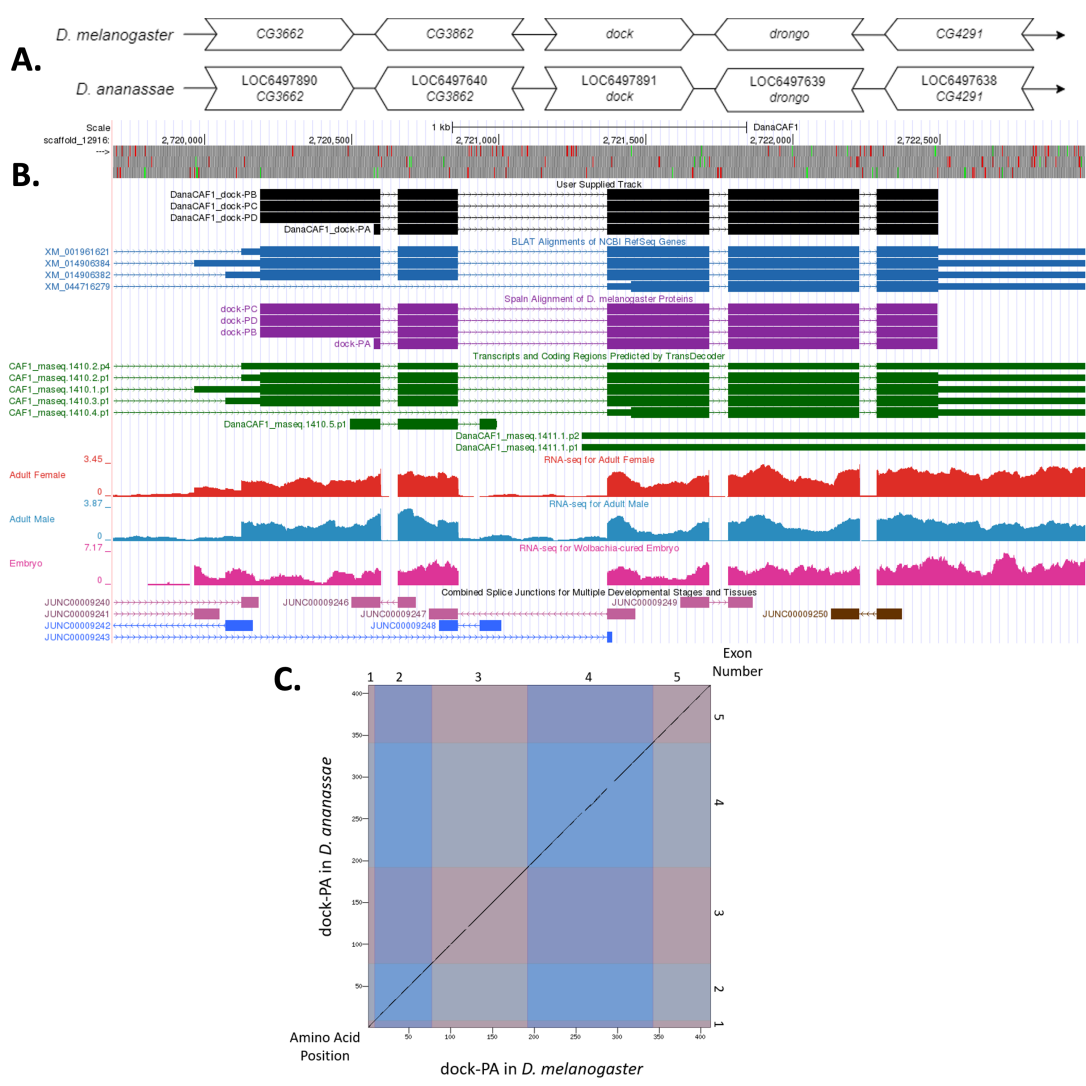
**
(A) Synteny comparison of the genomic neighborhoods for *dock* in *Drosophila melanogaster* and *D. ananassae* .** Thin underlying arrows indicate the DNA strand within which the gene– *dock* –is located in *D. melanogaster* (top) and *D. ananassae* (bottom). The thin arrows pointing to the right indicate that *dock* is on the positive (+) strand in *D. ananassae* and *D. melanogaster* . The wide gene arrows pointing in the same direction as *dock* are on the same strand relative to the thin underlying arrows, while wide gene arrows pointing in the opposite direction of *dock* are on the opposite strand relative to the thin underlying arrows. White gene arrows in *D. ananassae* indicate orthology to the corresponding gene in *D. melanogaster* . Gene symbols given in the *D. ananassae* gene arrows indicate the orthologous gene in *D. melanogaster* , while the locus identifiers are specific to *D. ananassae* . **(B) Gene Model in GEP UCSC Track Data Hub** (Raney et al., 2014). The coding-regions of *dock* in *D. ananassae* are displayed in the User Supplied Track (black); coding CDSs are depicted by thick rectangles and introns by thin lines with arrows indicating the direction of transcription. Subsequent evidence tracks include BLAT Alignments of NCBI RefSeq Genes (dark blue, alignment of Ref-Seq genes for *D. ananassae* ), Spaln of *D. melanogaster* Proteins (purple, alignment of Ref-Seq proteins from *D. melanogaster* ), Transcripts and Coding Regions Predicted by TransDecoder (dark green), RNA-Seq from Adult Females, Adult Males, and Wolbachia-cured Embryos (red, light blue, and pink, respectively); alignment of Illumina RNA-Seq reads from *D. ananassae* ), and Splice Junctions Predicted by regtools using *D. ananassae* RNA-Seq (SRP006203, SRP007906, PRJNA257286, PRJNA388952). Splice junctions shown have a minimum read-depth of 10 with 10-49, 100-499, 500-999 supporting reads in blue, pink, and brown, respectively. **(C) Dot Plot of dock-PA in *D. melanogaster* ( *x* -axis) vs. the orthologous peptide in *D. ananassae* ( *y* -axis).** Amino acid number is indicated along the left and bottom; CDS (exon) number is indicated along the top and right, and CDSs are also highlighted with alternating colors. Line breaks in the dot plot indicate mismatching amino acids at the specified location between species.

## Description

**Table:** 

*This article reports a predicted gene model generated by undergraduate work using a structured gene model annotation protocol defined by the Genomics Education Partnership (GEP; thegep.org) for Course-based Undergraduate Research Experience (CURE). The following information in this box may be repeated in other articles submitted by participants using the same GEP CURE protocol for annotating Drosophila species orthologs of Drosophila melanogaster genes in the insulin signaling pathway.* "In this GEP CURE protocol students use web-based tools to manually annotate genes in non-model *Drosophila* species based on orthology to genes in the well-annotated model organism fruitfly *Drosophila melanogaster* . The GEP uses web-based tools to allow undergraduates to participate in course-based research by generating manual annotations of genes in non-model species (Rele et al., 2023). Computational-based gene predictions in any organism are often improved by careful manual annotation and curation, allowing for more accurate analyses of gene and genome evolution (Mudge and Harrow 2016; Tello-Ruiz et al., 2019). These models of orthologous genes across species, such as the one presented here, then provide a reliable basis for further evolutionary genomic analyses when made available to the scientific community.” (Myers et al., 2024). “The particular gene ortholog described here was characterized as part of a developing dataset to study the evolution of the Insulin/insulin-like growth factor signaling pathway (IIS) across the genus *Drosophila* . The Insulin/insulin-like growth factor signaling pathway (IIS) is a highly conserved signaling pathway in animals and is central to mediating organismal responses to nutrients (Hietakangas and Cohen 2009; Grewal 2009).” (Myers et al., 2024). “The product of gene *dreadlocks* ( *dock* , FBgn0010583) is involved in several cellular functions, including axon guidance (Garrity et al., 1996; Hing et al., 1999; Schmucker et al., 2000; Stevens and Jacobs 2002; Weng et al., 2011), myoblast fusion during muscle fiber formation (Kaipa et al., 2013), regulation of intercellular bridges in germline cells during gametogenesis (Stark et al., 2021), and negative regulation of insulin receptor signaling pathway (Wu et al., 2011; Willoughby et al., 2017). A *dock* transcript was first isolated and sequenced in *Drosophila melanogaster* in a screen for P-element insertions leading to R cell projection defects (Garrity et al., 1996). *dock* encodes a protein that contains three N-terminal SH3 domains and one C-terminal SH2 domain known to bind to specific motifs and serve as binding adapters (Garrity et al., 1996). Its regulation of photoreceptor axon guidance in *Drosophila* occurs through its interaction with InR (Rao et al., 1998; Song et al., 2003; Rao 2005). The dock protein plays a role in negatively regulating the insulin signaling pathway by facilitating the dephosphorylation of InR through recruitment of the ER-localized form of protein tyrosine phosphatase PTP61F, a function that is also observed in its mammalian ortholog *Nck* (Wu et al., 2011; Buszard et al., 2013).” (Bicanovsky et al., 2024). “ *D* . *ananassae* (NCBI:txid7217) is part of the *melanogaster* species group within the subgenus *Sophophora* of the genus *Drosophila* (Sturtevant 1939; Bock and Wheeler 1972). It was first described by Doeschall (1858). *D. ananassae* is circumtropical (Markow and O'Grady 2005; https://www.taxodros.uzh.ch, accessed 1 Feb 2023), and often associated with human settlement (Singh 2010). It has been extensively studied as a model for its cytogenetic and genetic characteristics, and in experimental evolution (Kikkawa 1938; Singh and Yadav 2015).” (Lawson et al., 2024).

We propose a gene model for the *D. ananassae* ortholog of the *D. melanogaster* *dreadlocks* ( *dock* ) gene. The genomic region of the ortholog corresponds to the uncharacterized protein XP_001961657.1 ( LOC6497891 ) in the May 2011 (Agencourt dana_caf1/DanaCAF1) Genome Assembly of *D. ananassae* ( GCA_000005115.1 ; Drosophila 12 Genomes Consortium et al., 2007). This model is based on RNA-Seq data from *D. ananassae* ( SRP006203 , SRP007906 , PRJNA257286 , PRJNA388952 ; Graveley et al., 2011) and *dock* in *D. melanogaster* using FlyBase release FB2023_03 ( GCA_000001215.4 ; Larkin et al., 2021; Gramates et al., 2022; Jenkins et al., 2022).

***Synteny***

The reference gene, *dock ,* occurs on chromosome 2L in *D. melanogaster* and is flanked upstream by *CG3662* and *CG3862* and downstream by *drongo* and *CG4291* . The *tblastn* search of *D. melanogaster* dock-PA (query) against the *D. ananassae* ( GCA_000005115.1 ) Genome Assembly (database) placed the putative ortholog of *dock* within scaffold_12916 at locus LOC6497891 ( XP_001961657.1 )— with an E-value of 5e-171 and a percent identity of 83.54%. Furthermore, the putative ortholog is flanked upstream by LOC6497890 ( XP_001961655.1 ) and LOC6497640 ( XP_001961656.1 ), which correspond to *CG3662* and *CG3862* in *D. melanogaster* (E-value: 0.0 and 0.0; identity: 85.71% and 89.21%, respectively, as determined by *blastp* ; Figure 1A ; Altschul et al., 1990). The putative ortholog of *dock* is flanked downstream by LOC6497639 ( XP_001961658.2 ) and LOC6497638 ( XP_001961659.2 ), which correspond to *drongo* and *CG4291* in *D. melanogaster* (E-value: 0.0 and 0.0; identity: 74.27% and 84.66%, respectively, as determined by *blastp* ). The putative ortholog assignment for *dock* in *D. ananassae* is supported by the following evidence: The genes surrounding the *dock* ortholog are orthologous to the genes at the same locus in *D. melanogaster* and local synteny is completely conserved, supported by results generated from *blastp* , so we conclude that LOC6497891 is the correct ortholog of *dock* in *D. ananassae* ( Figure 1A ).

***Protein Model***

*dock* in *D. ananassae* has four mRNA isoforms. *dock-RB* , *dock-RC* , and *dock-RD* are translated into identical proteins, and *dock-RA* is distinct in that it has a shorter first CDS ( Figure 1B ). All the RNA isoforms contain five CDSs in *D. melanogaster* . Relative to the ortholog in *D. melanogaster* , the protein isoform count is conserved ( Figure 1B ). There is some evidence for a novel isoform that is shorter than the others as predicted in the RefSeq genes track (XM_044716279), however, given the low support for the splice junction (20 reads) we default to the protocol's assumptions (Rele et al., 2023) that isoform structure is conserved relative to *D. melanogaster* unless there is strong evidence to the contrary, thus we are not annotating a novel isoform. The sequence of dock-PD in *D. ananassae* has 85.41% identity (E-value: 0.0) with the protein-coding isoform dock-PD in *D. melanogaster* , as determined by *blastp* ( Figure 1C ). Coordinates of this curated gene model (dock-PA, dock-PD, dock-PC, dock-PB) are stored by NCBI at GenBank (accessions BK064611 , BK064612 , BK064613 , and BK064614 ). These data are also archived in the CaltechDATA repository (see “Extended Data” section below).

## Methods

Detailed methods including algorithms, database versions, and citations for the complete annotation process can be found in Rele et al. (2023). Briefly, students use the GEP instance of the UCSC Genome Browser v.435 (https://gander.wustl.edu; Kent WJ et al., 2002; Navarro Gonzalez et al., 2021) to examine the genomic neighborhood of their reference IIS gene in the *D. melanogaster* genome assembly (Aug. 2014; BDGP Release 6 + ISO1 MT/dm6). Students then retrieve the protein sequence for the *D. melanogaster* reference gene for a given isoform and run it using *tblastn* against their target *Drosophila* species genome assembly on the NCBI BLAST server (https://blast.ncbi.nlm.nih.gov/Blast.cgi; Altschul et al., 1990) to identify potential orthologs. To validate the potential ortholog, students compare the local genomic neighborhood of their potential ortholog with the genomic neighborhood of their reference gene in *D. melanogaster* . This local synteny analysis includes at minimum the two upstream and downstream genes relative to their putative ortholog. They also explore other sets of genomic evidence using multiple alignment tracks in the Genome Browser, including BLAT alignments of RefSeq Genes, Spaln alignment of *D. melanogaster* proteins, multiple gene prediction tracks (e.g., GeMoMa, Geneid, Augustus), and modENCODE RNA-Seq from the target species. Detailed explanation of how these lines of genomic evidenced are leveraged by students in gene model development are described in Rele et al. (2023). Genomic structure information (e.g., CDSs, intron-exon number and boundaries, number of isoforms) for the *D. melanogaster* reference gene is retrieved through the Gene Record Finder (https://gander.wustl.edu/~wilson/dmelgenerecord/index.html; Rele et al *.,* 2023). Approximate splice sites within the target gene are determined using *tblastn* using the CDSs from the *D. melanogaste* r reference gene. Coordinates of CDSs are then refined by examining aligned modENCODE RNA-Seq data, and by applying paradigms of molecular biology such as identifying canonical splice site sequences and ensuring the maintenance of an open reading frame across hypothesized splice sites. Students then confirm the biological validity of their target gene model using the Gene Model Checker (https://gander.wustl.edu/~wilson/genechecker/index.html; Rele et al., 2023), which compares the structure and translated sequence from their hypothesized target gene model against the *D. melanogaster* reference gene model. At least two independent models for a gene are generated by students under mentorship of their faculty course instructors. Those models are then reconciled by a third independent researcher mentored by the project leaders to produce the final model. Note: comparison of 5' and 3' UTR sequence information is not included in this GEP CURE protocol (Gruys et al., 2025).

## Data Availability

Description: A GFF, FASTA, and PEP of the model. Resource Type: Model. DOI: https://doi.org/10.22002/5rcbb-mpv03
